# A Lower Level of Forced Expiratory Volume in 1 Second Is a Risk Factor for All-Cause and Cardiovascular Mortality in a Japanese Population: The Takahata Study

**DOI:** 10.1371/journal.pone.0083725

**Published:** 2013-12-13

**Authors:** Yoko Shibata, Sumito Inoue, Akira Igarashi, Keiko Yamauchi, Shuichi Abe, Yasuko Aida, Keiko Nunomiya, Masamichi Sato, Hiroshi Nakano, Kento Sato, Takako Nemoto, Tomomi Kimura, Tetsu Watanabe, Tsuneo Konta, Makoto Daimon, Yoshiyuki Ueno, Takeo Kato, Takamasa Kayama, Isao Kubota

**Affiliations:** 1 Department of Cardiology, Pulmonology and Nephrology, School of Medicine, Yamagata University, Yamagata City, Yamagata, Japan; 2 Global Center of Excellence Program Study Group, School of Medicine, Yamagata University, Yamagata City, Yamagata, Japan; University Heart Center, Germany

## Abstract

Chronic obstructive pulmonary disease is a known risk factor for cardiovascular death in Western countries. Because Japan has a low cardiovascular death rate, the association between a lower level of forced expiratory volume in 1 s (FEV_1_) and mortality in Japan’s general population is unknown. To clarify this, we conducted a community-based longitudinal study. This study included 3253 subjects, who received spirometry from 2004 to 2006 in Takahata, with a 7-year follow-up. The causes of death were assessed on the basis of the death certificate. In 338 subjects, airflow obstruction was observed by spirometry. A total of 127 subjects died. Cardiovascular death was the second highest cause of death in this population. The pulmonary functions of the deceased subjects were significantly lower than those of the subjects who were alive at the end of follow-up. The relative risk of death by all causes, respiratory failure, lung cancer, and cardiovascular disease was significantly increased with airflow obstruction. The Kaplan–Meier analysis showed that all-cause and cardiovascular mortality significantly increased with a worsening severity of airflow obstruction. After adjusting for possible factors that could influence prognosis, a Cox proportional hazard model analysis revealed that a lower level of FEV_1_ was an independent risk factor for all-cause and cardiovascular mortality (per 10% increase; hazard ratio [HR], 0.89; 95% confidence interval [CI], 0.82–0.98; and HR, 0.72; 95% CI, 0.61–0.86, respectively). In conclusion, airflow obstruction is an independent risk factor for all-cause and cardiovascular death in the Japanese general population. Spirometry might be a useful test to evaluate the risk of cardiovascular death and detect the risk of respiratory death by lung cancer or respiratory failure in healthy Japanese individuals.

## Introduction

The significant association of chronic obstructive pulmonary disease (COPD) on all-cause and cardiovascular death has been reported, and a lower level of forced expiratory volume in 1 s (FEV_1_) has been reported as a risk for all-cause and cardiovascular mortality in Western countries [1-3]. COPD and cardiovascular disease share the same risk factors such as cigarette smoking, systemic inflammation, and aging. Therefore, cardiovascular disease is a representative comorbidity in patients with COPD [4].

In Japan, the prevalence of COPD is estimated to be at least 8.6% in the general population aged ≥40 years [5]. Because the prevalence of COPD in other countries was reported to be approximately 10%, there seems to be no great difference regarding to the prevalence of COPD among different ethnicities [4]. However, for Japanese COPD patients, their cardiovascular death rate was estimated to be not as high as that of patients in Western countries. In fact, as Japanese people generally have a healthy lifestyle such as a habitual intake of low calorie foods, the Japanese population is usually leaner and has a lower risk of cardiovascular events than that of Western countries [6]. 

Recently, a high prevalence of COPD among Japanese patients in cardiology clinics [7] and the development of atherosclerosis in Japanese COPD patients [8] have been reported. These suggest that even for the Japanese, cardiovascular disease is a significant comorbidity in COPD patients. Moreover, the number of COPD patients in Japan is estimated to be 5 million [5]. However, only 170 thousand are diagnosed with and treated for COPD [9]. Therefore, the true cause of death in Japanese COPD patients including undiagnosed individuals has not been assessed, and the association between airflow obstruction and cardiovascular death has not been elucidated in ethnicities with a low cardiovascular death rate, such as the Japanese. To assess these issues, analyses regarding the cause of death among subjects with airflow obstruction, mainly consisting of COPD patients, in the Japanese general population cohort should be useful [10-16].

We hypothesized that subjects with airflow obstruction in the general Japanese population have a higher cardiovascular death rate and that a lower level of FEV_1_ is a risk factor for a cardiovascular death. The aim of this study was to evaluate the cause of death in subjects with airflow obstruction and the association of a lower level of FEV_1_ with cardiovascular death in a Japanese population that participated in an annual health check in the town of Takahata, Yamagata, Japan.

## Methods

### Study population

This study was part of the Molecular Epidemiological Study utilizing the Regional Characteristics of 21^st^ Century Centers of Excellence (COE) Program and the Global COE Program in Japan [13,15]. The ethics committee of the Yamagata University School of Medicine approved the study, and all participants provided written informed consent.

The study was based on an annual community health check, in which all residents aged 40 years or older in the northern Japanese town of Takahata were invited to participate. From 2004 to 2006, 1579 men and 1941 women (a total of 3520 subjects) were enrolled in this study. A total of 267 subjects were excluded from the analysis because of insufficient spirometry data or objection to follow-up. The data for a total of 3253 subjects (1500 men; 1753 women) were entered into the final statistical analysis [13,15]. Subjects used a self-reported questionnaire to document their medical history, smoking habits, current medication use, and clinical symptoms. Eleven men and 10 women were receiving therapy for pulmonary diseases. However, information on the precise diagnosis of their diseases and prescription usage was not available in the database. The lifetime consumption of cigarette smoke was assessed using pack-years (daily number of cigarette packs × years). To investigate the association between spirometric values and prognosis, a follow-up survey was performed annually until the end of 2010 [17,18]. The median follow-up period was 6.2 years. During this follow-up period, 127 subjects died. Subjects who moved during the follow-up period were identified by residence transfer documents (n = 37). The cause of death was determined by reviewing death certificates through the end of 2010. The death code (International Classification of Diseases, 10^th^ Revision) and the date and place of death were reviewed [17,18].

### Measurements

Blood pressure (systolic and diastolic) was measured using a mercury sphygmomanometer. Blood samples were taken from the antecubital vein of subjects who had been fasting, and the samples were immediately transferred to chilled tubes. Spirometric parameters (forced vital capacity [FVC] and FEV_1_) were measured using standard techniques, with subjects performing FVC maneuvers on a CHESTAC-25 part II EX instrument (Chest Corp, Tokyo, Japan), according to the guidelines of the Japanese Respiratory Society (JRS) [19]. A bronchodilator was not administered prior to spirometry. The highest value from at least 3 FVC maneuvers from each subject was used for the analysis. After excluding those subjects with inadequate data, the results were assessed by 2 pulmonary physicians who visually inspected the flow-volume curves, as defined by the JRS criteria [19]. An airflow obstruction was defined as FEV_1_/FVC < 0.70, instead of FEV_1_/VC < 5^th^ percentile of the predicted value [20], because FEV_1_/FVC < 0.7 is more common. Of 2095 never-smokers and 1158 current/past smokers, 127 (6.1%) and 211 (18.2%) subjects had airflow obstruction, respectively.

### Statistical analysis

For continuous variables, data are presented as mean values (SD). Student’s *t* test for parametric data and the Mann–Whitney *U* test for non-parametric data were used to analyze the differences between the 2 groups. The χ^2^ test was performed to evaluate the differences in proportion. A Kaplan–Meier method with log-rank test was performed to examine the relationship between airflow obstruction and all-cause and cardiovascular mortality. Cox proportional hazard analyses were performed to assess if the FEV_1_% predicted or the tertiles of FEV_1_% predicted were associated with the all-cause and cardiovascular mortality, independent of the other possible confounding factors. The results of the Cox proportional hazard analyses are presented as hazard ratios (HR) with 95% confidence intervals (CI). A statistical significance was inferred for two-sided *P* values < 0.05. For the evaluation of the goodness of fit, the overall model fit for sequential models was compared using the Akaike information criterion (AIC), which takes into account both the statistical goodness of fit and the number of parameters required to achieve this particular degree of fit, by imposing a penalty for increasing the number of parameters. AIC values were obtained by using the following formula: AIC = -2 × (maximum log likelihood) + 2 × (number of parameters) [21,22]. Lower AIC values indicate better models. In practice, the difference in an AIC of 1 or greater between the models means a superior fit for the model that has a lower AIC [23-25]. A receiver operator characteristics (ROC) curve analysis, as well as area under the curve (AUC) was used as a measure of the predictive accuracy of FEV_1_ on all-cause death and cardiovascular death. In addition, we calculated the net reclassification improvement (NRI) and the integrated discrimination improvement (IDI) in order to measure the quantity of improvement for the correct reclassification and sensitivity according to the addition of FEV_1_ to the model [26]. Statistical analyses were performed using JMP version 9 software (SAS Institute Inc, Cary, NC) or R 3.0.2 with additional packages (Rcmdr, Epi, pROC and PredictABEL).

## Results

### Comparison of characteristics between subjects who were alive and subjects who had died by the end of the study follow-up

During the median 6.2-year follow-up, 127 subjects died. The differences in age, sex, body mass index (BMI), smoking status, and pulmonary functions, as well as the proportion receiving therapy for respiratory diseases, hypertension, cardiac diseases, stroke, and dyslipidemia, between the subjects who had died and those who were alive at the end of the follow-up period, are summarized in Table 1. 

**Table 1:** Comparison of characteristics between subjects who were alive and subjects who had died by the end of the study follow-up.

	subjects who were alive at the end of follow-up period (n = 3126)	subjects who had died by the end of follow-up period (n = 127)	*P*
age	61.9 (10.3)	71.6 (8.2)	<0.0001
male	45.1%	71.7%	<0.0001
BMI	23.5 (3.2)	22.6 (3.5)	0.001
Smoking status, %, never/current/past	64.8/18.9/16.3	52.8/23.6/23.6	0.017
Pack-years #	9.5 (19.1)	16.5 (25.7)	0.0002
FVC% predicted	98.8 (14.4)	95.1 (19.3)	0.0051
FEV_1_% predicted	98.0 (16.2)	92.0 (23.2)	<0.0001
FEV_1_/FVC	78.7 (7.6)	75.0 (11.0)	<0.0001
airflow obstruction	9.80%	24.40%	<0.0001
respiratory disease therapy	0.64%	0.79%	0.843
hypertension therapy	34.4%	48.0%	0.002
cardiac disease therapy	7.4%	14.2%	0.011
stroke therapy	1.76%	4.72%	0.041
dyslipidemia therapy	12.2%	3.9%	0.001

#Pack-years were available for 2811 of those subjects who were alive and 113 of those who had died.

Data are expressed as mean (SD) or percentage.

FVC, forced vital capacity; FEV_1_, forced expiratory volume in 1 s.

At the end of the follow-up period, the subjects who had died were significantly older, more likely male, less obese, and more likely a current or past smoker, as well as had more cigarette smoke exposure, than that of those subjects who were alive. The FVC, FEV_1_, and FEV_1_/FVC were significantly lower and the percentage of airflow obstruction was higher in the subjects who had died than that of those who were alive. In addition, the subjects who had died during the follow-up period were receiving more hypertension, cardiac disease, and stroke treatments and less dyslipidemia treatments, compared to that of the subjects were alive at the end of the study. Respiratory disease therapies were not administered to many subjects in this study population. There was no difference regarding the proportion of subjects receiving respiratory therapy between those who were alive and those who had died during the follow-up period.

### Cause of death for the subjects with and without airflow obstruction

As summarized in Table 2, in this study group, the top 3 causes of death were neoplasms (total, 35.4%; lung cancer, 9.4%; and other organ neoplasm, 26.0%), cardiovascular disease (26.8%), and respiratory failure (21.2%). Of the total 3253 subjects, 338 subjects had airflow obstruction. In subjects without airflow obstruction, the top 3 causes of death were neoplasms (total, 32.3%; lung cancer, 2.1%; and other organ neoplasms, 30.2%), cardiovascular disease (26.0%), and respiratory failure (22.9%), while the top 3 causes in subjects with airflow obstruction were respiratory failure (32.3%), neoplasms (total, 29.0%; lung cancer, 16.1%; and other organ neoplasms, 12.9%), and cardiovascular disease (29.0%). With cardiovascular disease, 16 deaths were related to myocardial infarction, specifically, 9 subjects without airflow obstruction and 7 with airflow obstruction. In this study population, in total, all-cause death rates were 3.90%, with 3.29% for subjects without airflow obstruction and 9.17% for subjects with airflow obstruction. Death rates related to all causes, respiratory failure, lung cancer, and cardiovascular disease were significantly higher for subjects with airflow obstruction than those without (Table 2). The myocardial infarction death rate was 0.49% in total; the myocardial infarction death rate for subjects with airflow obstruction (2.07%) was higher than that of subjects without airflow obstruction (0.31%). As shown in Table 3, relative risks of death from all causes, respiratory failure, lung cancer, and cardiovascular disease, in particular, myocardial infarction, were significantly elevated with the presence of airflow obstruction.

**Table 2:** Cause of death relative to airflow obstruction in this study.

Cause of death	all subjects (n = 3253)	subjects without airflow obstruction (n = 2915)	subjects with airflow obstruction (n = 338)
Respiratory failure	27 (21.2%, 0.83%)	17 (17.7%, 0.58%)	10 (32.3%, 2.96%*)
Lung cancer	12 (9.4%, 0.37%)	7 (2.1%, 0.07%)	5 (16.1%, 1.48%*)
Cardiovascular disease	34 (26.8%, 1.05%)	25 (26.0%, 0.86%)	9 (29.0%, 2.66%*)
Other organ neoplasms	33 (26.0%, 1.01%)	29 (30.2%, 0.99%)	4 (12.9%, 1.18%)
Others	21 (16.5%, 0.65%)	18 (18.8%, 0.62%)	3 (9.7%, 0.89%)
All causes	127 (100%, 3.90%)	96 (100%, 3.29%)	31 (100%, 9.17%*)

* : P < 0.05 versus subjects without airflow obstruction (χ^2^ test)

Data are expressed as number of deaths (percentage for the number of all-cause deaths in each category and the percentage for the total number of subjects in each category).

**Table 3:** Increase in relative risk of death with airflow obstruction.

Cause of death	relative risk	95% confidence interval	*P*
All causes	2.78	1.89–4.11	<0.0001
Respiratory failure	5.07	2.34–10.99	<0.0001
Lung cancer	6.16	1.97–19.30	0.0004
Cardiovascular disease	3.10	1.46–6.60	0.002
Myocardial infarction	6.70	2.51 - 17.90	<0.0001

References were the mortality risk of the subjects without airflow obstruction.

### Kaplan–Meier and Cox proportional hazard analyses

**Figure 1:**
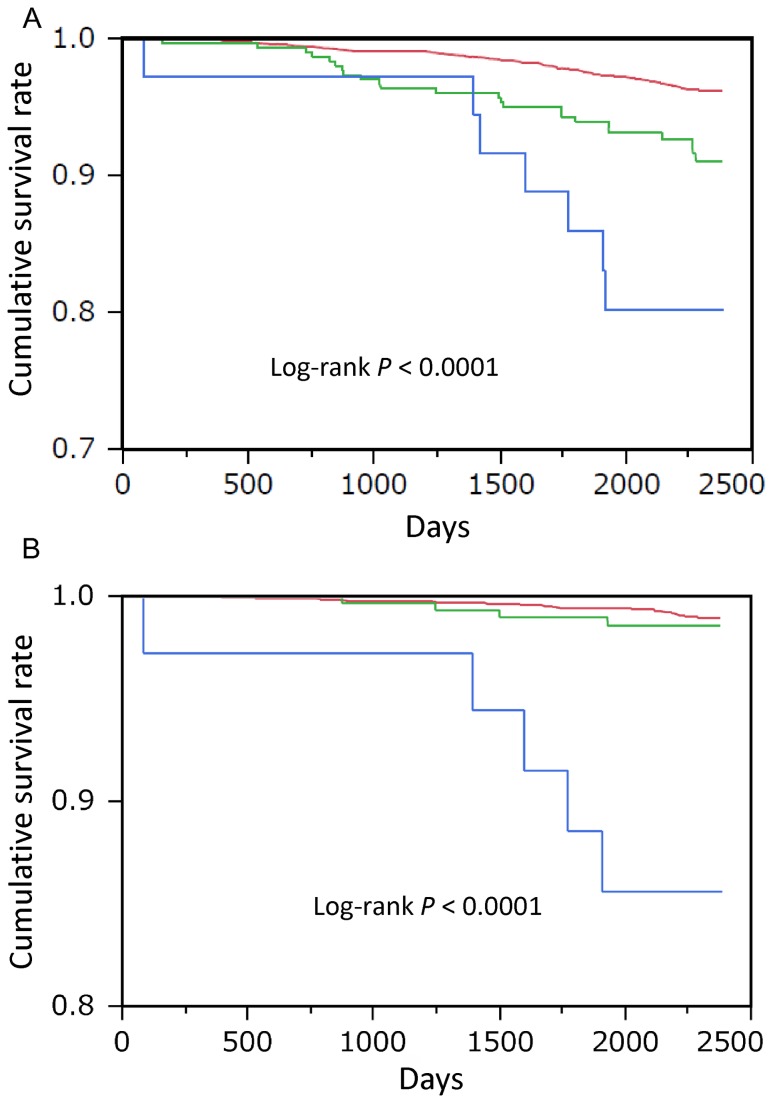
Kaplan–Meier survival curve for all-cause and cardiovascular mortality according to the severity of airflow obstruction using the cutoff of FEV_1_% predicted 50%.

**Figure 2:**
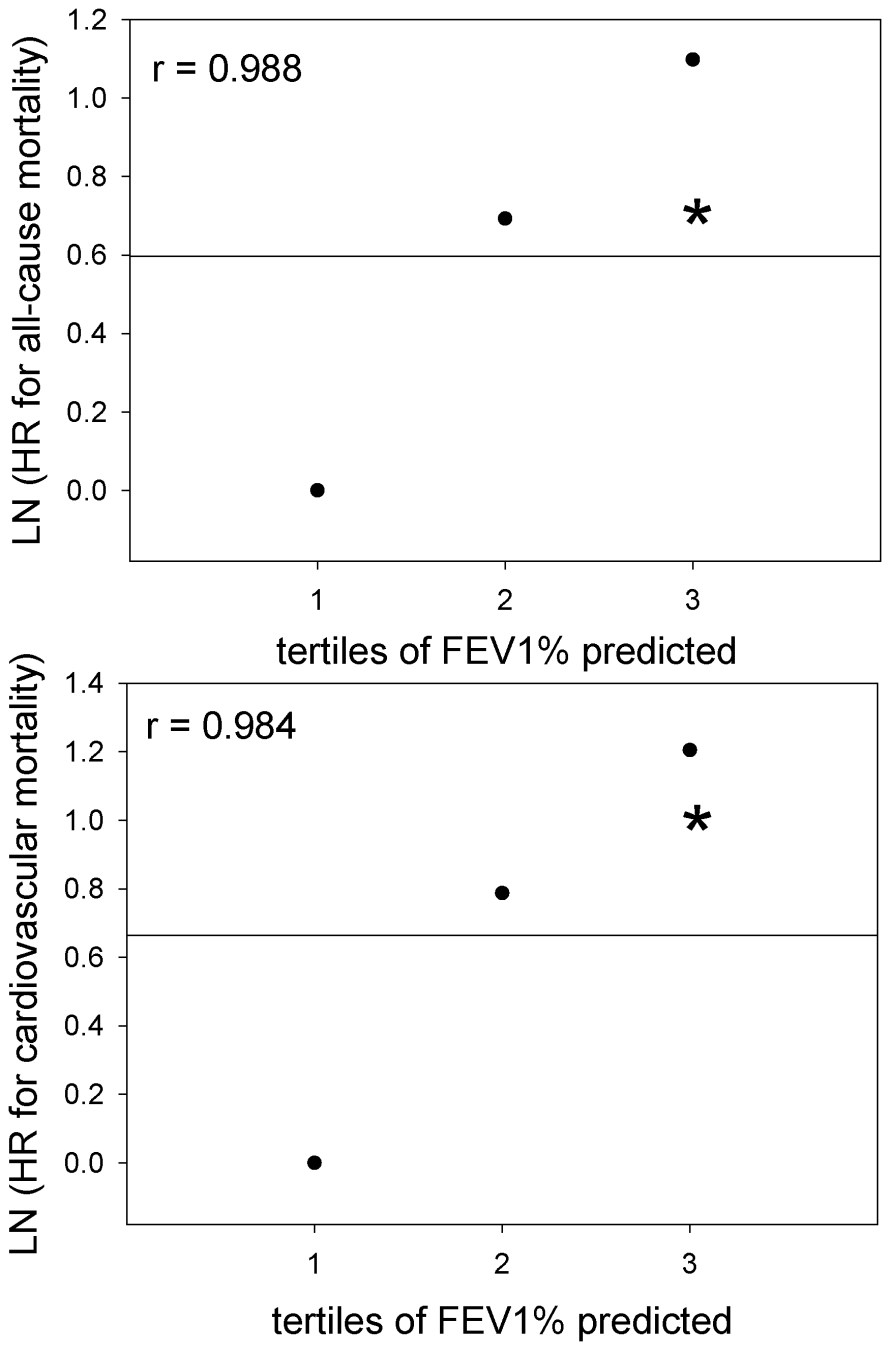
Linear elevation of the hazard ratio for all-cause mortality and cardiovascular mortality relative to the tertiles of FEV_1_ % predicted. The graphs show the linear elevation of the hazard ratio (HR) for all-cause (upper) and cardiovascular (lower) mortality according to a decrease in FEV_1_% predicted. Because the hazard ratio is an exponential value of a coefficient of the regression analyses, linearity of the coefficient could be assessed by a natural-log (LN) HR. First tertile (n = 1072; all-cause death = 33; cardiovascular death = 5), FEV_1_% predicted > 104.7%; second tertile (n = 1105; all-cause death = 34; cardiovascular death = 9), FEV_1_% predicted of ≤ 104.7% and >92.0%; and third tertile (n = 1076; all-cause death = 60; cardiovascular death = 20), FEV_1_% predicted ≤ 92.0%. Data were adjusted for age, sex, body mass index, pack-years, systolic blood pressure, alanine aminotransferase, serum creatinine, hemoglobin A1c, triglyceride, and total cholesterol.

Figure 1A and 1B show the survival rates for all-cause and cardiovascular mortality according to the severity of airflow obstruction. The survival rate was significantly reduced with a worsening severity of airflow obstruction. Because the subjects with airflow obstruction in this study were older and more likely male and had more cigarette-smoke exposure than that of subjects without obstruction, a Cox proportional hazard analysis was performed to adjust for possible confounding factors (Table 4). We applied representative variates that could influence the prognosis of the subjects, such as age, gender, body mass index (BMI), lifetime cigarette consumption (pack-years), hypertension (systolic blood pressure), liver function (alanine aminotransferase), renal function (serum creatinine), diabetes (hemoglobin A1c), and dyslipidemia (triglyceride and total cholesterol) to this analysis. A lower level of FEV_1_% predicted was a significant risk factor for all-cause mortality, independent of the other possible confounding factors (Table 4). In univariate Cox proportional hazard analyses, a lower level of FEV_1_ was a risk factor for mortality of cardiovascular disease and respiratory failure but not for death by lung cancer (Table 5). In multivariate Cox proportional hazard analyses, a lower level of FEV_1_% predicted was a significant risk factor for cardiovascular mortality but not for death by lung cancer or respiratory failure, independent of the other possible confounding factors (Table 6). As shown in Figure 2, the relationship between the tertiles of FEV_1_% predicted and the logarithmically transformed value of those hazard ratios was linear. In order to examine whether model fit and discrimination improves with the addition of FEV_1_ to the basic predictors, age and sex, we evaluated the difference between AIC and AUC in the ROC curves, as well as the improvement of NRI and IDI between two models with or without FEV1% predicted as a predictive parameter (Tables 7 and 8). The differences in AIC with the Cox proportional analyses between the two models were 3.0 and 14.7 for all-cause (Table 7) and cardiovascular mortality (Table 8), respectively. The AUC in the ROC curve for cardiovascular mortality was significantly greater in the model including FEV_1_% predicted than those not including it, while the AUC in the ROC curve for all-cause mortality in the model including FEV_1_% predicted did not significantly differ from those not including it. Furthermore, the inclusion of FEV_1_% predicted in the model with age and sex for the prediction of cardiovascular death improved the NRI and IDI values, suggesting effective reclassification and discrimination, although the inclusion of FEV_1_% predicted in the model with age and sex for all-cause death prediction did not.

**Table 4:** A lower level of FEV_1_ is an independent risk factor for all-cause mortality.

	HR	95% CI	*P*
age (per 1-year increase)	1.11	1.08–1.14	<0.0001
male (versus female)	2.25	1.42–3.61	0.0006
BMI (per 1 kg/m^2^ increase)	0.95	0.89–1.01	0.09
pack-years (per 1 pack-year increase)	1.00	0.99–1.01	0.493
systolic BP (per 1-mmHg increase)	1.00	0.98–1.01	0.681
ALT (per 1 IU/L increase)	1.01	0.99–1.02	0.334
sCr (per 1 mg/dL increase)	1.18	0.56–1.76	0.576
HbA1c (per 1% increase)	1.04	0.79–1.29	0.773
TG (per 1 mg/dL increase)	1.00	1.00–1.00	0.989
TC (per 1 mg/dL increase)	1.00	0.99–1.00	0.415
FEV_1_% predicted (per 10% increase)	0.89	0.82–0.98	0.019

Cox proportional hazard analysis.

ALT, alanine aminotransferase; BMI, body mass index; BP, blood pressure; CI, confidence interval; Cig., cigarette; FEV_1_, forced expiratory volume in 1 s; HbA1c, hemoglobin A1c; HR, hazard ratio; sCr, serum creatinine; TC, total cholesterol; TG, triglyceride.

**Table 5:** A lower level of FEV_1_ is a risk factor for mortality of cardiovascular disease and respiratory failure but not for death by lung cancer.

cause of mortality	HR	95% CI	*P*
cardiovascular disease	0.63	0.54–0.74	<0.0001
lung cancer	1.01	0.73–1.44	0.956
respiratory failure	0.74	0.61–0.90	0.0038

Cox proportional hazard analysis (unadjusted). HRs for per 10% increase in FEV_1_% predicted are shown.

CI, confidence interval; FEV1, forced expiratory volume in 1 s; HR, hazard ratio

**Table 6:** A lower level of FEV_1_ is an independent risk factor for cardiovascular mortality but not for death by lung cancer or respiratory failure.

cause of mortality	HR	95% CI	*P*
cardiovascular disease	0.72	0.61–0.86	0.0002
lung cancer	1.07	0.78–1.51	0.700
respiratory failure	0.83	0.69–1.01	0.066

Cox proportional hazard analysis (multivariate). Data were adjusted for age, gender, BMI, pack-years, systolic BP, ALT, sCr, HbA1c, TG, and TC. HRs for per 10% increase in FEV_1_% predicted are shown.

CI, confidence interval; FEV_1_, forced expiratory volume in 1 s; HR, hazard ratio.

**Table 7:** Statistics for model fit and improvement with the addition of FEV_1_% predicted on the prediction of all-cause mortality.

	Age + Sex	Age + Sex + FEV_1_% predicted	*P*	Difference
AIC	1875.4	1872.4	—	3.0
AUC of ROC curve	0.794	0.779	0.2455	—
NRI (95% CI)	—	0.11 (-0.07 to 0.28)	0.2265	—
IDI (95% CI)	—	0.003 (-0.002 to 0.008)	0.2517	—

AIC, Akaike information criterion; AUC, area under the curve; CI, confidence interval; FEV_1_, forced expiratory volume in 1 s; IDI, integrated discrimination improvement; NRI, net reclassification improvement; ROC, receiver operator characteristics.

**Table 8:** Statistics for model fit and improvement with the addition of FEV_1_% predicted on the prediction of cardiovascular mortality.

	Age + Sex	Age + Sex + FEV_1_% predicted	*P*	Difference
AIC	505.8	491.2	—	14.7
AUC of ROC curve	0.773	0.794	0.0019	—
NRI (95% CI)	—	0.342 (0.01 to 0.68)	0.0455	—
IDI (95% CI)	—	0.019 (0.002 to 0.04)	0.0329	—

AIC, Akaike information criterion; AUC, area under the curve; CI, confidence interval; FEV_1_, forced expiratory volume in 1 s; IDI, integrated discrimination improvement; NRI, net reclassification improvement; ROC, receiver operator characteristics

Graphs show the survival curve in all-cause (A) and cardiovascular (B) mortality relative to the severity of airflow obstruction using the cutoff of FEV_1_% predicted 50%. The red curve indicates the survival rate of subjects without airflow obstruction. The green indicates an FEV_1_% predicted ≥ 50 airflow obstruction. The blue indicates an FEV_1_% predicted < 50 airflow obstruction. The survival rate was significantly reduced according to the elevation of severity of airflow obstruction.

*; *P* < 0.05 versus 1^st^ tertile.

A lower level of FEV_1_% predicted was not an independent risk factor for death by lung cancer or respiratory failure with the other confounding factors (Table 6). The lung cancer deaths for subjects with mild (n = 138; FEV_1_% predicted ≥ 80), moderate (n = 164; 50 ≤ FEV_1_% predicted < 80), and severe (n = 36; FEV_1_% predicted < 50) airflow obstruction were 3 (2.17%), 2 (1.22%), and 0 (0.00%), respectively. Even though smoking status was included in the analyses of Tables 4 and 6, smoking status was not a significant variate for all-cause, cardiovascular, lung cancer or respiratory failure mortality, and the inclusion of smoking status as a variate in these analyses did not change the significance of FEV_1_ for all-cause and cardiovascular mortality (data not shown).

## Discussion

In this study, we demonstrated that mortalities by all causes, cardiovascular, respiratory failure, and lung cancer were significantly increased in subjects with airflow obstruction, compared to those without airflow obstruction in the Japanese general population. The Kaplan–Meier method and the Cox proportional hazard analyses revealed that a lower level of FEV_1_ was a significant risk factor for all-cause and cardiovascular mortality in this population. Because the AIC in the model of the Cox proportional hazard analysis, including age, sex, and FEV_1_% predicted as parameters, was lower than that of model not including FEV_1_% predicted and because the difference in the AICs between the two models was greater than 1, the addition of FEV_1_ to the parameters seemed to improve the fitness of the model [21,22,25]. Further analyses revealed that the inclusion of FEV_1_ to the basic predictors, age and sex, significantly improved the prediction of cardiovascular death, but not all-cause death, with the model (Tables 7 and 8).

The reports from Western countries have demonstrated a significant association between a lower level of pulmonary function and all-cause and cardiovascular mortality. In the Copenhagen City Heart Study (662 males and 2048 females, from 1976 through 1986), a lower level of FEV_1_% predicted, FVC% predicted, and FEV_1_/FVC were significant risk factors of mortality for never-smokers in a general population, independent of age, sex, BMI, alcohol consumption, school education, diabetes, heart disease, and bronchial asthma [2]. In the Renfrew and Paisley prospective population study (7058 men and 8353 women, from the year 1972 through 1991), significant trends of increasing risk with a diminishing FEV_1_ for all causes of death were observed in both sexes after adjustment for age, cigarette smoking, diastolic BP, cholesterol concentration, BMI, and social class [1]. Corresponding relative hazard ratios of mortality from ischemic heart disease for the lowest fifth of the FEV_1_ distribution were 1.56 (1.26–1.92) in men and 1.88 (1.44–2.47) in women. In the National Health and Nutrition Examination Survey Epidemiologic Follow-up Study (n = 1861, from 1971 through 1992), individuals with the lowest FEV_1_ quintile had the highest risk of cardiovascular death (relative risk, 5.65; 95% CI, 2.26–14.13) [3]. To our knowledge, a lower level of FEV_1_ has not been demonstrated as a risk for all-cause and cardiovascular mortality among an Asian population. Compared to individuals in Western countries, Japanese individuals are generally thinner, and the prevalence of ischemic heart disease is lower [6]. In addition, the surveys demonstrating the association between pulmonary function and cardiovascular mortality were performed over 20 years ago. Due to the differences in ethnicities and the recent advanced therapies for hypertension and dyslipidemia, such as statins and angiotensin receptor blockers, this association has not yet been established for Japanese individuals. The current 7-year study showed that a lower level of FEV_1_ was an independent risk factor for all-cause and cardiovascular mortality in the Japanese general population. Our findings confirmed the significance of pulmonary function on prognosis, similar to past studies in a Western population.

In the Lung Health Study, subjects with mild-to-moderate airflow obstruction (n = 5887) were followed for 14.5 years; for 23%, the cause of death was a cardiovascular disease [27]. In the Hokkaido COPD Cohort Study (n = 279), Nishimura and their colleagues reported that 1.8% of COPD patients have cardiovascular comorbidity, and for 12%, the cause of death was a cardiovascular disease during the 5-year follow-up [28]. In the present study, the proportion of cardiovascular deaths for all-cause mortality (29%) seemed to be higher than that of the Hokkaido COPD Cohort Study. In the Hokkaido COPD Cohort Study, the enrolled subjects were definitely diagnosed in medical institutions and may be optimally treated, including for cardiovascular event risk factors, such as hypertension, diabetes, and dyslipidemia. However, many undiagnosed COPD patients in the general population are unaware of their condition until they develop some symptoms such as shortness of breath. In the present study, most subjects were healthy and had no apparent respiratory symptoms because this cohort was based on the study of an annual health check in a general population. Initially, less than 1% of the subjects in this study population were receiving therapy for respiratory diseases. Even after spirometric examination in the present study, most subjects with airflow obstruction may not have received optimal treatment for their respiratory illness because of a lack of airway symptoms. 

The Evaluation of COPD Longitudinally to Identify Predictive Surrogate Endpoints (ECLIPSE) study demonstrated that the prevalence of cardiovascular diseases was increased in COPD patients, independent of the severity of airflow obstruction [29]. In the present study, the cardiovascular death was highly skewed in the subjects with severe airflow obstruction (Figure 1). In the Understanding Potential Long-Term Impacts on Function with Tiotropium (UPLIFT) study, the use of tiotropium reduced the adverse events of cardiovascular diseases, such as congestive heart failure and myocardial infarction, suggesting that optimal treatment for COPD may reduce cardiovascular events [30]. The higher rate of cardiovascular death in this study may be related to the low proportion of subjects receiving treatment for a respiratory illness, as compared with a hospital-based cohort (Hokkaido COPD Cohort).

Interestingly, this study demonstrated the importance of lung function for the evaluation of the prognosis of subjects who received an annual health check. As shown in Table 4, the Cox proportional hazard analysis demonstrated that the all-cause mortality was more influenced by impaired pulmonary function than that of hypertension, diabetic status, dyslipidemia, and liver and kidney dysfunction in this study population. For cardiovascular death, other confounding factors such as gender, BMI, pack-years, systolic blood pressure, ALT, sCr, HbA1c, TG, and TC were not independent risk factors (data not shown). Therefore, spirometry should be considered and performed for Japanese individuals, along with other routine examinations including BP measurements or other clinical parameters, such as blood glucose and blood lipids.

It is widely known that COPD is a risk for lung cancer. However, the Cox proportional hazard analysis failed to show that a lower level of FEV_1_ is an independent risk for lung cancer death, although the crude death rate of lung cancer was higher for the subjects with airflow obstruction. In this study, lung cancer deaths were not observed in the subjects with severe airflow obstruction (see the *Results* section). In the BODE COPD observational study, 215 of the 2507 patients with COPD developed lung cancer, and the lung cancer incidence was lower in patients with a high severity of airflow obstruction [31]. As Cosio and Saetta reviewed, smokers who evade the development of severe COPD by immune system suppression would have a higher incidence of lung cancer than that of smokers who develop severe COPD [32].

As shown in Tables 4 and 6, cigarette smoking was not an independent risk factor for all-cause and cardiovascular mortality in this study population. It is well known that a lower respiratory function is associated with cigarette smoking, and cigarette smoking is a major risk factor for many diseases including cardiovascular diseases. In this study, information regarding cigarette smoking was obtained from a self-reported questionnaire, and some former smokers may have incorrectly answered their smoking history as never-smokers. However, we could not know how accurate this information regarding their smoking history was. In addition, among 338 subjects with airflow obstruction, 127 subjects (37.6%) were never-smokers. This number of never-smokers with airflow obstruction cannot be ignored and may create difficulty with respect to smoking habit or pack-years as a significant risk factor for all-cause and cardiovascular mortality in this study.

This study had several limitations. First, this study collected the baseline information with a 1-time measurement; laboratory and spirometric data usually show day-to-day variation. Second, nonfatal events were not included in this study; that omission might underestimate the association between a lower level of pulmonary function and outcome. Third, information on a final diagnosis of airflow obstruction was not available. In a Japanese adult population, the prevalence of COPD and bronchial asthma was estimated to be about 8.6% and 3%, respectively. These 2 are suggested as the major causes of airflow obstruction in this population. Fourth, sampling bias may exist because employees of public institutions and many companies did not participate in this research because those individuals are annually followed in different health programs. We cannot deny the possibility that differences in jobs or lifestyles between participants and non-participants may have affected the results of this research. Finally, post-bronchodilator spirometric values were not measured in this study. Because sufficient medicines and usual medical devices could not be prepared outside the hospital where the health-checks were performed, bronchodilator use was difficult to assess in the present study. Using post-bronchodilator spirometry substantially reduce the prevalence of subjects with persistent airflow obstruction such as COPD. Although the airflow obstruction observed in the present study may not always persistent, the results obtained in this study indicated that a pre-bronchodilator FEV_1_ at a lower level was associated with an increased risk of all-cause and cardiovascular mortality.

In conclusion, a lower level of FEV_1_ was a significant risk factor for all-cause and cardiovascular mortality in the Japanese general population. Even in ethnicities with a low cardiovascular event rate such as the Japanese, cardiovascular disease is an important comorbidity in subjects with airflow obstruction, namely undiagnosed COPD. In Japan, the recent diffusion rate for spirometry in clinic was less than 20% because of its complicated procedure for general physicians [33]. If spirometry were widely used by not only respiratory physicians, but also many other physicians including cardiologists, FEV_1_ could be a useful indicator for the early detection of high-risk subjects and the prevention of premature death by cardiovascular events.

## References

[1] Hole DJ, Watt GC, Davey-Smith G, Hart CL, Gillis CR et al. (1996) Impaired lung function and mortality risk in men and women: findings from the Renfrew and Paisley prospective population study. BMJ 313: 711-716; discussion: 8819439. 8819439 10.1136/bmj.313.7059.711 PMC2352103

[2] Lange P, Nyboe J, Appleyard M, Jensen G, Schnohr P (1990) Spirometric findings and mortality in never-smokers. J Clin Epidemiol 43: 867-873. doi:10.1016/0895-4356(90)90070-6. PubMed: 2213076. 2213076

[3] Sin DD, Wu L, Man SF (2005) The relationship between reduced lung function and cardiovascular mortality: a population-based study and a systematic review of the literature. Chest 127: 1952-1959. doi:10.1378/chest.127.6.1952. PubMed: 15947307. 15947307

[4] GOLD Global Initiative for Chronic. Obstructive Lung Disease. Global Strategy for the Diagnosis, Management and Prevention of Chronic Obstructive Pulmonary Disease (updated 2011).

[5] Fukuchi Y, Nishimura M, Ichinose M, Adachi M, Nagai A et al. (2004) COPD in Japan: the Nippon COPD Epidemiology study. Respirology 9: 458-465. doi:10.1111/j.1440-1843.2004.00637.x. PubMed: 15612956. 15612956

[6] WHO (2007) World Health. Statistics.

[7] Wada H, Nakano Y, Nagao T, Osawa M, Yamada H et al. (2010) Detection and prevalence of chronic obstructive pulmonary disease in a cardiovascular clinic: evaluation using a hand held FEV(1)/FEV(6) meter and questionnaire. Respirology 15: 1252-1258. doi:10.1111/j.1440-1843.2010.01854.x. PubMed: 20920134. 20920134

[8] Iwamoto H, Yokoyama A, Kitahara Y, Ishikawa N, Haruta Y et al. (2009) Airflow limitation in smokers is associated with subclinical atherosclerosis. Am J Respir Crit Care Med 179: 35-40. doi:10.1164/rccm.200804-560OC. PubMed: 18931335. 18931335

[9] Nagai A (2013) Epidemiology and Socie-economic burden. Tokyo: Medical Review co., ltd. 161 pp.

[10] Aida Y, Shibata Y, Osaka D, Abe S, Inoue S et al. (2011) The relationship between serum uric acid and spirometric values in participants in a health check: the Takahata study. Int J Med Sci 8: 470-478. PubMed: 21850198. 21850198 10.7150/ijms.8.470 PMC3156995

[11] Kishi H, Shibata Y, Osaka D, Abe S, Inoue S et al. (2011) FEV6 and FEV1/FEV6 in Japanese participants of the community-based annual health check: the Takahata study. Intern Med 50: 87-93. doi:10.2169/internalmedicine.50.4276. PubMed: 21245630. 21245630

[12] Nemoto T, Shibata Y, Osaka D, Abe S, Inoue S et al. (2011) Impact of cigarette smoking on maximal expiratory flows in a general population: the Takahata study. Intern Med 50: 2547-2555. doi:10.2169/internalmedicine.50.5948. PubMed: 22041355. 22041355

[13] Nunomiya K, Shibata Y, Abe S, Inoue S, Igarashi A et al. (2013) Hyperhomocysteinaemia predicts the decline in pulmonary function in healthy male smokers. Eur Respir J 42: 18-27. doi:10.1183/09031936.00066212. PubMed: 23143543. 23143543

[14] Osaka D, Shibata Y, Abe S, Inoue S, Tokairin Y et al. (2010) Relationship between habit of cigarette smoking and airflow limitation in healthy Japanese individuals: the Takahata study. Intern Med 49: 1489-1499. doi:10.2169/internalmedicine.49.3364. PubMed: 20686279. 20686279

[15] Sato M, Shibata Y, Abe S, Inoue S, Igarashi A et al. (2013) Retrospective analysis of the relationship between decline in FEV1 and abdominal circumference in male smokers: The Takahata Study. Int J Med Sci 10: 1-7. doi:10.7150/ijms.5003. PubMed: 23288999. 23288999 PMC3534871

[16] Shibata Y, Watanabe T, Osaka D, Abe S, Inoue S et al. (2011) Impairment of pulmonary function is an independent risk factor for atrial fibrillation: the Takahata study. Int J Med Sci 8: 514-522. PubMed: 21897765. 21897765 10.7150/ijms.8.514 PMC3167177

[17] Daimon M, Konta T, Oizumi T, Karasawa S, Kaino W et al. (2012) Higher plasma renin activity is a risk factor for total mortality in older Japanese individuals: the Takahata study. Metabolism 61: 504-511. doi:10.1016/j.metabol.2011.08.004. PubMed: 22001336. 22001336

[18] Konta T, Kudo K, Sato H, Ichikawa K, Ikeda A et al. (2013) Albuminuria is an independent predictor of all-cause and cardiovascular mortality in the Japanese population: the Takahata study. Clin Exp Nephrol: ([MedlinePgn:]) PubMed: 23345069. 10.1007/s10157-013-0770-3 23345069

[19] The Committee of Pulmonary Physiology JRS (2004) Guidelines for Pulmonary Function Tests: Spirometry, flow-volume curve, diffusion capacity of the lung. Tokyo: Medical Review Co., Ltd. p. 56.

[20] Pellegrino R, Viegi G, Brusasco V, Crapo RO, Burgos F et al. (2005) Interpretative strategies for lung function tests. Eur Respir J 26: 948-968. doi:10.1183/09031936.05.00035205. PubMed: 16264058. 16264058

[21] Steyerberg E (2009) Clinical Prediction Models: A Practical Approach to Development, Validation and Updating. New York: Springer.

[22] Tangri N, Stevens LA, Griffith J, Tighiouart H, Djurdjev O et al. (2011) A predictive model for progression of chronic kidney disease to kidney failure. JAMA 305: 1553-1559. doi:10.1001/jama.2011.451. PubMed: 21482743. 21482743

[23] Burnham K, Anderson DR (2002) Model Selection and Multimodel Inference: A Practical Information-Theoretic Approach. New York: Springer.

[24] Wagenmakers EJ, Farrell S (2004) AIC model selection using Akaike weights. Psychon Bull Rev 11: 192-196. doi:10.3758/BF03206482. PubMed: 15117008. 15117008

[25] Yamaoka K, Nakagawa T, Uno T (1978) Application of Akaike's information criterion (AIC) in the evaluation of linear pharmacokinetic equations. J Pharmacokinet Biopharm 6: 165-175. PubMed: 671222. 671222 10.1007/BF01117450

[26] Pencina MJ, D'Agostino RB Sr., D'Agostino RB Jr., Vasan RS (2008) Evaluating the added predictive ability of a new marker: from area under the ROC curve to reclassification and beyond. Stat Med 27: 157-212; discussion: 17569110. 17569110 10.1002/sim.2929

[27] Anthonisen NR, Skeans MA, Wise RA, Manfreda J, Kanner RE et al. (2005) The effects of a smoking cessation intervention on 14.5-year mortality: a randomized clinical trial. Ann Intern Med 142: 233-239. doi:10.7326/0003-4819-142-4-200502150-00005. PubMed: 15710956. 15710956

[28] Nishimura M, Makita H, Nagai K, Konno S, Nasuhara Y et al. (2012) Annual change in pulmonary function and clinical phenotype in chronic obstructive pulmonary disease. Am J Respir Crit Care Med 185: 44-52. doi:10.1164/rccm.201106-0992OC. PubMed: 22016444. 22016444

[29] Agusti A, Calverley PM, Celli B, Coxson HO, Edwards LD et al. (2010) Characterisation of COPD heterogeneity in the ECLIPSE cohort. Respir Res 11: 122 PubMed: 20831787. 20831787 10.1186/1465-9921-11-122 PMC2944278

[30] Tashkin DP, Celli B, Senn S, Burkhart D, Kesten S et al. (2008) A 4-year trial of tiotropium in chronic obstructive pulmonary disease. N Engl J Med 359: 1543-1554. doi:10.1056/NEJMoa0805800. PubMed: 18836213. 18836213

[31] de Torres JP, Marín JM, Casanova C, Cote C, Carrizo S et al. (2011) Lung cancer in patients with chronic obstructive pulmonary disease-- incidence and predicting factors. Am J Respir Crit Care Med 184: 913-919. doi:10.1164/rccm.201103-0430OC. PubMed: 21799072. 21799072

[32] Cosio MG, Saetta M (2012) Evasion of COPD in smokers: at what price? Eur Respir J 39: 1298-1303. doi:10.1183/09031936.00135711. PubMed: 22005915. 22005915

[33] Suwabe A (2008) Respiratory diseases (in Japanese). J Jpn Soc Int Med 97: 2927-2935. 10.2169/naika.97.2927 19320103

